# Development of Natural Killer Cell–Drug Conjugates via Membrane-Installed Liposomes for Pancreatic Cancer Treatment

**DOI:** 10.34133/bmr.0285

**Published:** 2025-12-09

**Authors:** Ashok Kumar Jangid, Chae Eun Lee, Minseon Ryu, Sungjun Kim, Kyobum Kim

**Affiliations:** Department of Chemical & Biochemical Engineering, Dongguk University, Seoul, Republic of Korea.

## Abstract

Adoptive cell-based therapy has emerged as an innovative method for cancer treatment, capitalizing on the innate cytotoxicity of immune cells to eliminate tumors. Although chimeric-antigen-receptor-modified T and natural killer (NK) cells have demonstrated significant therapeutic potential, their clinical translation is hindered by the complex nature of genetic engineering, high production costs, and risks of severe immune-related adverse effects. Addressing these barriers, we present a biomaterial-based approach to engineering NK cells, entirely bypassing the need for genetic modification. Initially, we systematically evaluated the surface modification of NK cells by employing a range of dibenzocyclooctyne (DBCO)–lipid biomaterials based on 1,2-distearoyl-*sn*-glycero-3-phosphoethanolamine (DSPE) lipid: (a) 2 linear structures with different polyethylene glycol (PEG) chain lengths (DSPE-PEG2k-DBCO and DSPE-PEG5k-DBCO), (b) a tadpole structure (DSPE-PEG2k-Di-PEG2k-DBCO), and (c) a branched structure (DSPE-PEG2k-HA-DBCO). The tadpole-shaped DSPE-PEG2k-Di-PEG2k-DBCO exhibited remarkable membrane anchoring, biocompatibility, and preservation of membrane integrity and facilitated the subsequent conjugation of gemcitabine-loaded liposomes (GLipo) through DBCO–azide click chemistry, as validated using fluorescence microscopy. The fabricated GLipo-NK cell–drug conjugates maintained native NK cell viability (>80%) and enabled targeted drug release at tumor sites. Our GLipo-modified NK cells showed superior in vitro cytotoxicity against MIA PaCa-2 pancreatic cancer cells, attributed to a synergistic interaction between immune synapse formation and innate NK-cell-mediated cytotoxicity. This strategy establishes a robust framework for the development of safe, scalable, and effective cell-based immunotherapies aimed at treating solid tumors.

## Introduction

Adoptive cell therapy has revolutionized cancer treatment by employing immune cells to target and eradicate tumor tissues [1,2]. Adoptive cell therapy leverages ex vivo cell surface modification techniques, such as introducing novel T-cell receptors or synthetic chimeric antigen receptors (CARs), to enhance effector cell functions. The inherent ability of immune cells to kill tumors, together with these membrane engineering approaches, enables high-affinity tumor recognition, prolonged immune activation, and augmented tumor-killing efficacy [3]. Currently, CAR-modified natural killer (CAR-NK) cells and CAR-T cells constitute the leading strategies to improve treatment outcomes against tumor cells. Nevertheless, CAR-T cell therapies encounter significant challenges, including intricate manufacturing processes, substantial costs, and adverse effects such as cytokine release syndrome, and graft-versus-host disease [4,5]. These constraints have driven substantial interest in CAR-NK cells, which offer distinct advantages such as inherent cytotoxicity, reduced cytokine release syndrome risk, and suitability for off-the-shelf allogeneic application [6,7]. Despite these promising features, CAR-NK cells frequently exhibit limited transfection efficiency, and unintended genetic modification may compromise their baseline anti-tumor properties. Consequently, there is a critical need to develop optimized, safe, and highly efficient surface modification methods for infusible NK cells to fully realize their therapeutic capacity [8].

To overcome the limitations associated with CAR-based cell surface manipulation, biomaterial-mediated ex vivo cell surface engineering of NK cells has emerged as a promising alternative [9,10]. This technique allows for rapid and efficient coating of NK cells, thereby eliminating the need for complex genetic modifications and facilitating immunological synapse-dependent cancer recognition [11,12]. Among the different types of biomaterials, lipid-based materials are advantageous due to their ability to anchor to the cell surface through hydrophobic interactions, offering nontoxic, stable attachment and presenting additional reactive moieties outward on the cell surface [6,7,13–17]. Furthermore, incorporating therapeutic drugs into administrative immune cells further enhances tumor regression outcomes. This innovative approach, known as the “cell–drug conjugate (CDC)” strategy, involves coupling chemo-drug moieties to immune cell surfaces, resulting in controlled and localized drug release within the tumor microenvironment (TME) while preserving the intrinsic cytotoxicity of effector cells [18–25]. However, the development of effective CDCs is often hindered by significant challenges, including the cytotoxicity of drug payloads to immune cells resulting from improper membrane immobilization and unintentional cytoplasmic infiltration, inadequate payload loading, and suboptimal drug release profiles [26–30].

**Fig. 1.**
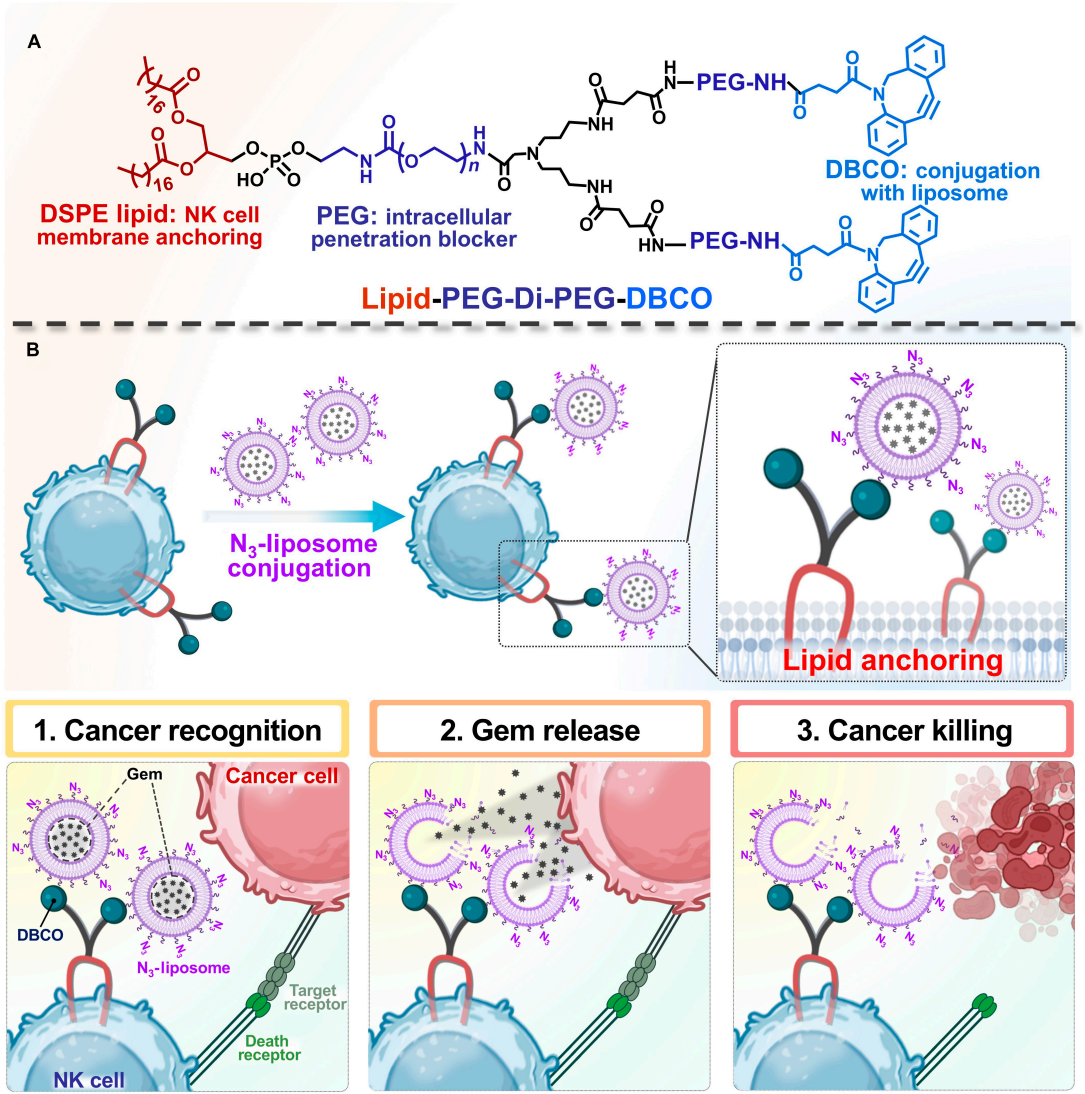
(A) Chemical structure of the synthesized tadpole dibenzocyclooctyne (DBCO)–lipid biomaterial for natural killer (NK) cell membrane anchoring and (B) illustration of NK cell surface modification using DBCO–lipid biomaterials, subsequent liposome conjugation on DBCO-NK cells, and anticancer mechanism of GLipo-NK cells. GLipo, gemcitabine-loaded liposomes; DSPE, 1,2-distearoyl-*sn*-glycero-3-phosphoethanolamine; PEG, polyethylene glycol; Gem, gemcitabine.

Therefore, our design strategy for effective CDC fabrication involved the lipid anchor-mediated installation of liposomes onto NK cell surfaces. Specifically, a sequential fabrication process was adopted: (a) modification of NK cell surfaces with a dibenzocyclooctyne (DBCO)–lipid conjugate (Fig. 1A) via hydrophobic interactions and (b) immobilization of gemcitabine (gem)-loaded liposomes (GLipo) on NK membranes through covalent bond formation using a straightforward click chemistry approach. Our DBCO–lipid conjugates provide (a) a nontoxic and efficient method for NK cell surface modification without disrupting the native anticancer functions of NK cells and (b) site-specific conjugation of GLipo onto NK membranes. In this study, we established ex vivo surface-engineered NK cells functionalized with GLipo as an innovative CDC platform for cancer therapy. The engineering process involved (a) optimized coating of NK cell surfaces with various DBCO–lipid conjugate materials via membrane anchoring (designated as DBCO-NK cells) and (b) conjugation with azide-modified GLipo (GLipo-NK cells). The GLipo-NK cells demonstrated enhanced anticancer efficacy via a sequential codelivery mechanism illustrated in Fig. 1B: (a) NK-cell-mediated cancer targeting through immunological synapse interactions, (b) localized gem release and activation of NK cells, and (c) improved tumor cell eradication. GLipo-NK cells preserved the inherent properties of NK cells and showed improved in vitro anticancer performance against MIA PaCa-2 pancreatic cancer cells through the integration of NK cytotoxicity and gem-induced apoptosis. Collectively, these results highlight that GLipo-NK represents a robust cancer immunotherapy platform and an efficient delivery system in comparison with immune cell therapy alone.

## Materials and Methods

### Chemical reagents for synthesis of DBCO–lipid conjugates

All lipid conjugates were prepared using DSPE (1,2-distearoyl-*sn*-glycero-3-phosphoethanolamine) as the lipid anchor. DSPE-PEG2k-amine (LP096005-2K), DSPE-PEG5k-amine (LP096005-5K), DSPE-PEG2k-azide (LP096006-2K), DBCO acid (BP-22625), and DBCO-amine (BP-22066) were obtained from Biopharma PEG Scientific Inc. (USA). 1-Ethyl-3-(3-(dimethylamino)propyl)carbodiimide (EDC; E7750-25G), *N*-hydroxysuccinimide (NHS; 130672-25G), *N*,*N*-bis(*N*′-Fmoc-3-aminopropyl)glycine potassium hemisulfate (Di-Fmoc-Gly; 14832), anhydrous dimethylformamide (DMF; 611903), 4-dimethylaminopyridine (DMAP; 611903), bis-polyethylene glycol amine 2000 (bis-PEG2k-amine; 14501), 1,2-dimyristoyl-*sn*-glycero-3-phosphocholine (DMPC) lipid (850345P), theophylline (PHL82668), and deuterated nuclear magnetic resonance (NMR) solvent were purchased from Sigma-Aldrich (USA). Hyaluronic acid (HA; HA60K-1) was supplied by Lifecore Biomedical, Inc. (USA). Gem (G0544) was acquired from Tokyo Chemical Industry (Tokyo, Japan). A Spectrum Spectra dialysis membrane tubing (132620) was purchased from Fisher Scientific (Sweden).

### Synthesis of DBCO–lipid conjugates for NK cell surface engineering

To optimize the presentation of DBCO on NK cell surfaces, we designed 4 distinct types of lipid-based membrane anchor materials: 2 linear structures with varying polyethylene glycol (PEG) chain lengths (DSPE-PEG2k-DBCO and DSPE-PEG5k-DBCO), a tadpole structure (DSPE-PEG2k-Di-PEG2k-DBCO), and a branched structure (DSPE-PEG2k-HA-DBCO). Each DBCO–lipid conjugate was synthesized using an EDC/NHS-mediated coupling reaction. For the linear constructs, DBCO acid was conjugated with DSPE-PEG2k-amine or DSPE-PEG5k-amine via coupling chemistry (Fig. S1). In summary, 13.8 mg of DBCO acid, 21.7 mg of EDC, and 21.78 mg of NHS were dissolved in 1 ml of anhydrous DMF and reacted for 30 min at room temperature (RT) under N_2_ protection. Next, 50 mg of DSPE-PEG2k-amine in 1 ml of DMF was added along with a catalytic amount of DMAP. The reaction was maintained at RT for 24 h. The resulting product was dialyzed (molecular weight cut-off [MWCO] 2 kDa) in deionized water (DW) for 3 d to remove unreacted impurities. The synthesis of DSPE-PEG5k-DBCO followed a similar procedure.

The branched biomaterial was synthesized through 2 consecutive reaction steps as shown in Fig. S2. In the first step, DSPE-PEG2k-amine was grafted onto the HA backbone, followed by reaction of DBCO-amine with the DSPE-PEG2k-HA bioconjugate. Specifically, 100 mg of HA and an excess of EDC and NHS were dissolved in 5 ml of phosphate-buffered saline (PBS) at pH 7.4 and stirred at RT for 3 h. Then, 34.3 mg of DSPE-PEG2k-amine in 2 ml of DMF was introduced together with a catalytic amount of DMAP. The reaction was maintained at RT for 48 h. The product was then dialyzed (MWCO 12 to 14 kDa) in DW for 3 d for purification and subsequently lyophilized. In the second step, 50 mg of DSPE-PEG2k-HA bioconjugate, 8 mg of EDC, and 7 mg of NHS were dissolved in 5 ml of PBS pH 7.4 and stirred at RT for 3 h. Afterward, 7 mg of DBCO-amine and 5 mg of DMAP dissolved in 5 ml of DMF were added to the reaction mixture. This mixture was stirred at RT for 48 h, dialyzed (MWCO 12 to 14 kDa) in DW for 2 d for purification, and finally lyophilized.

**Fig. 2.**
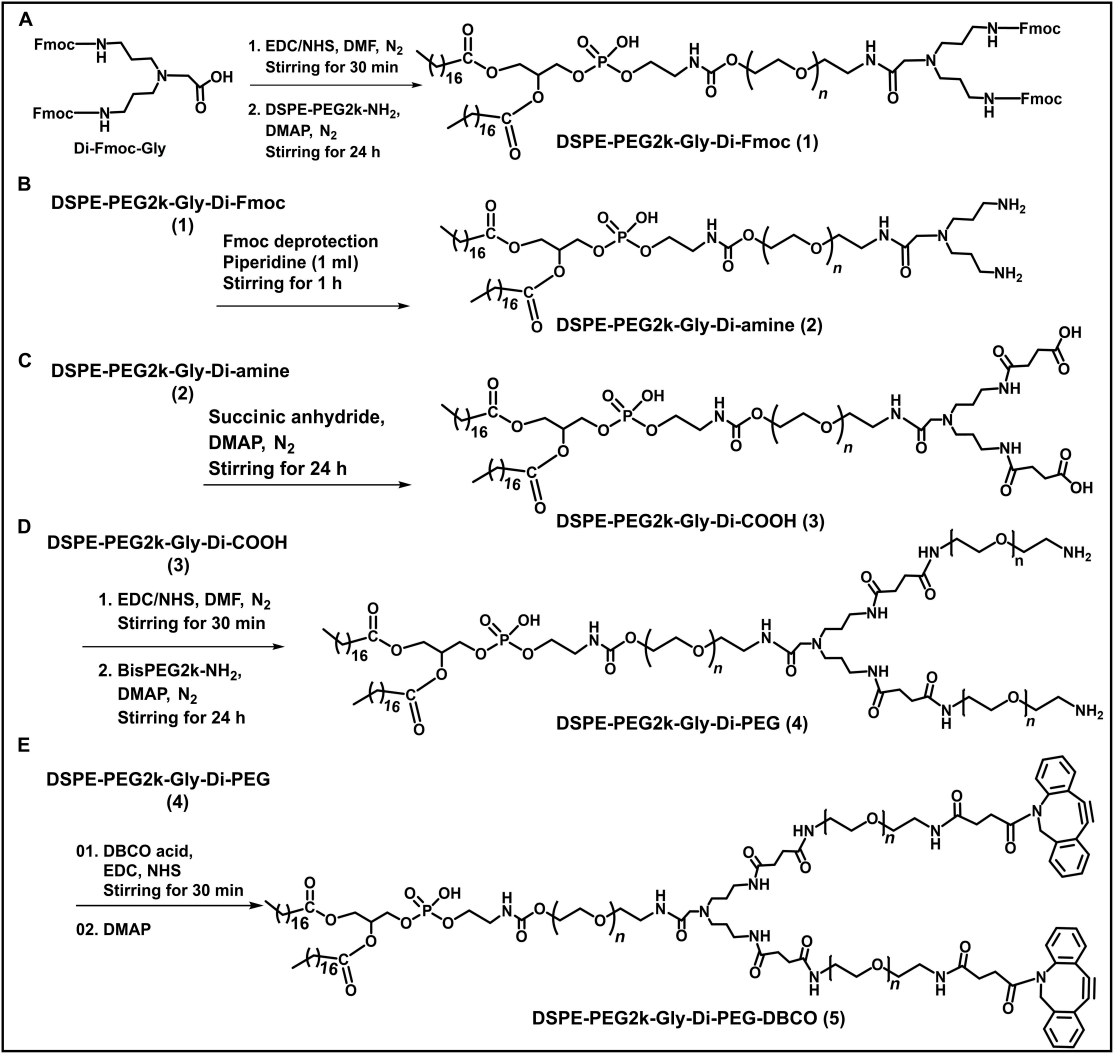
Synthesis route steps of the tadpole-style DSPE-PEG2k-Di-PEG2k-DBCO biomaterial. (A) DSPE-PEG2k-amine conversion into DSPE-PEG2k-di-Fmoc via *N*,*N*-bis(*N*′-Fmoc-3-aminopropyl)glycine potassium hemisulfate (Di-Fmoc-Gly) conjugation using the 1-ethyl-3-(3-(dimethylamino)propyl)carbodiimide (EDC)/*N*-hydroxysuccinimide (NHS) coupling reaction, (B) DSPE-PEG2k-di-Fmoc conversion into DSPE-PEG2k-di-amine followed by Fmoc deprotection, (C) DSPE-PEG2k-di-amine conversion into DSPE-PEG2k-di-COOH followed by a succinylation reaction mechanism, (D) DSPE-PEG2k-di-COOH conversion into DSPE-PEG2k-di-PEG via bis-PEG-amine conjugation using the EDC/NHS coupling reaction, and (E) synthesis of the DSPE-PEG2k-Di-PEG2k-DBCO biomaterial via DBCO acid conjugation with DSPE-PEG2k-di-PEG using the EDC/NHS coupling reaction. DMF, dimethylformamide; DMAP, 4-dimethylaminopyridine.

The DSPE-PEG2k-Di-PEG2k-DBCO biomaterial (featuring a tadpole structure) was synthesized as described in our previous report, with some modifications (Fig. 2) [15,16]. In brief, 82.7 mg of Di-Fmoc-Gly, 24.7 mg of EDC, and 30.9 mg of NHS were dissolved in anhydrous DMF and allowed to react for 30 min under N_2_ protection. Subsequently, 200 mg of DSPE-PEG2k-amine was introduced along with a catalytic amount of DMAP. The reaction mixture was stirred at RT for 24 h under a N_2_ atmosphere. Piperidine (1 ml) was then added to remove the Fmoc group. The resulting DSPE-PEG2k-Gly-Di-amine was precipitated using cold diethyl ether and dialyzed (MWCO 2 kDa) in DW for 1 d to eliminate impurities. In the following step, DSPE-PEG2k-Gly-Di-amine was converted to DSPE-PEG2k-Gly-Di-COOH by reaction with succinic anhydride in the presence of DMAP for 24 h under N_2_ protection. The reaction mixture was subsequently dialyzed (MWCO 2 kDa) in DW for 2 d for further purification, followed by lyophilization to yield a solid powdered product. Subsequently, 100 mg of DSPE-PEG2k-Gly-Di-COOH together with excess amounts of 15 mg of EDC and 13 mg of NHS were dissolved in anhydrous DMF and reacted for 30 min under N_2_ protection. Following this, 140 mg of bis-PEG2k-amine was added along with a catalytic quantity of 5 mg of DMAP. The reaction mixture was stirred at RT for 24 h under N_2_. The mixture was then dialyzed (MWCO 3.5 kDa) in DW for 3 d to remove unreacted impurities, after which it was lyophilized to obtain a solid powdered DSPE-PEG2k-Di-PEG2k intermediate. For the final step, the DSPE-PEG2k-Di-PEG2k intermediate was reacted with DBCO acid. Specifically, 12.9 mg of DBCO acid, 28.3 mg of EDC, and 24.3 mg of NHS were dissolved in 1 ml of anhydrous DMF and allowed to react for 30 min at RT under N_2_. Thereafter, 100 mg of the DSPE-PEG2k-Di-PEG2k intermediate was added together with a catalytic quantity of 5 mg of DMAP. The reaction mixture was stirred at RT for 24 h. This mixture was then dialyzed (MWCO 2 kDa) in DW for 3 d to remove unreacted impurities. All synthesized biomaterials were characterized by ^1^H NMR analysis (500-MHz Fourier transform NMR spectrometer, Bruker, Germany). The NMR spectra were plotted and analyzed using the MestReNova 6.0.0–5475 software.

### Cell cultivation

All cells were obtained from the American Type Culture Collection (ATCC, USA). Minimum essential medium alpha (MEMα; 1967785), fetal bovine serum (FBS; 16000044), and 12.5% horse serum (16050122) were purchased from Gibco (USA). Penicillin–streptomycin solution (30-002-CI), 89% Dulbecco’s modified Eagle’s medium (DMEM; 10-013-CV), and FBS (35-015-CV) were purchased from Corning (USA). NK-92mi cells (ATCC, CRL-2408) were cultured in complete growth medium consisting of MEMα (Gibco, 1967785), supplemented with 12.5% FBS (Gibco, 16000044), 12.5% horse serum (Gibco, 16050122), 1% penicillin–streptomycin solution (Corning, 30-002-CI), 0.1 mM β-mercaptoethanol (Sigma-Aldrich, 21985023), 0.2 mM folic acid (Sigma-Aldrich, F8758), and 0.2 mM *myo*-inositol (Sigma-Aldrich, I5125). MIA PaCa-2 cells (ATCC, CRL-1420) were cultured in a growth medium composed of 89% DMEM (Corning, 10-013-CV), 10% FBS (Corning, 35-015-CV), and 1% penicillin–streptomycin solution (Corning, 30-002-CI). All cell cultures were maintained at 37 °C with 5% CO_2_ and 95% humidity.

### NK cell surface coating using DBCO–lipid conjugates

To systematically evaluate the influence of polymer architecture on NK cell surface engineering, 4 different DBCO–lipid conjugates were utilized to coat NK cellular membranes: 2 linear types (DSPE-PEG2k-DBCO and DSPE-PEG5k-DBCO), a tadpole-like structure (DSPE-PEG2k-Di-PEG2k-DBCO), and a branched structure (DSPE-PEG2k-HA-DBCO). In summary, NK cells (5 × 10^5^ cells) were incubated with 100 μl of each coating formulation (0.75 mg/ml in MEMα) at RT for 30 min and then washed twice. To visualize DBCO modification on NK membranes, 10 μM BP Fluor 488 Picolyl Azide (BroadPharm, USA, BP-25551) was subsequently added and incubated at RT for 30 min. After an additional 2 washes, surface fluorescence was monitored with fluorescence microscopy (Ti-E System, Nikon, Japan), and images were processed using the ImageJ software (National Institutes of Health, USA).

### Characterization of DBCO-NK cells

The intrinsic properties of DBCO-NK cells, obtained using a tadpole-structured DBCO–lipid conjugate, were thoroughly characterized. Initially, the expression levels of Fas ligand (FasL) and tumor-necrosis-factor-related apoptosis-inducing ligand (TRAIL), key mediators of anticancer function, were examined at the NK cell surface. NK or DBCO-NK cells (5 × 10^5^ cells) were stained with APC-conjugated FasL antibody (BD Biosciences, USA, 564262) and TRAIL antibody (BD Biosciences, USA, 563642) at 4 °C for 30 min. After antibody binding, cells were washed 3 times with cold PBS and analyzed using flow cytometry (Beckman Coulter, USA). In addition, cytokine secretion following antigenic stimulation was assessed. NK or DBCO-NK cells (1.5 × 10^5^ cells) were stimulated with 0.5 μg/ml lipopolysaccharide (LPS) (from *Escherichia coli* O26:B6, Sigma-Aldrich, L8274) at 37 °C for 24 h. After stimulation, supernatants were collected and interferon-γ (IFN-γ) concentrations were measured with an enzyme-linked immunosorbent assay kit (PeproTech, USA, 900-T27) in accordance with the manufacturer’s instructions.

Subsequently, NK cell viability post-DBCO modification was examined. NK or DBCO-NK cells were placed in 96-well plates at 1.0 × 10^5^ cells per well and incubated at 37 °C for 24 h. Viability was measured by the CellTiter-Blue assay (Promega, USA, G8081) following the manufacturer’s protocol. The resulting fluorescence intensity (Ex/Em 560/590 nm) was detected using an ultraviolet (UV)–visible spectrophotometer (iD3, SpectraMax, USA).

Furthermore, the intrinsic cytotoxicity of NK cells was quantified by determining the percentage of cancer cell death within a co-culture system. Both NK and DBCO-NK cells were incubated with MIA PaCa-2 cells (1.0 × 10^4^ cells per well) at an effector-to-target ratio of 2:1. Following 24 h of incubation, suspended cells were centrifuged and supernatants were carefully removed. Cytotoxicity was then evaluated using the WST-1 assay (DoGenBio, Republic of Korea, Ez-3000), performed according to the manufacturer’s protocol. The percentage of cancer cell death was determined using the following equation: Cancer cell death (%) = [1 − (experimental value − effector control)/(target cell spontaneous control)] × 100 (%), where the experimental value corresponds to co-cultures of NK or DBCO-NK cells with MIA PaCa-2 cells, effector control denotes cultures of NK or DBCO-NK cells alone (without cancer cells), and target cell spontaneous control indicates MIA PaCa-2 cells cultured alone (without NK cells).

### Liposome fabrication and characterization

GLipo were prepared by employing a thin-film hydration method. To optimize the formulation, several ratios of DMPC lipid and DSPE-PEG2k-azide (1:2, 2:3, 1:1, 4:3, and 2:1) were evaluated to achieve a consistent particle size distribution. Specifically, 100 μl of DMPC lipid (10 mg/ml) and 50 μl of DSPE-PEG2k-azide (10 mg/ml), each dissolved in chloroform, were combined in a 5-ml glass vial. The solvent was removed by evaporation under N_2_, resulting in the formation of a thin lipid film. This thin film was rehydrated using 1 ml of PBS (pH 7.4) containing 1 mg/ml of gem, followed by sonication with a probe tip sonicator for 2 min at a 20% duty cycle using a 5-s on/off setting. Free gem not encapsulated within the liposomes was separated using an Amicon Ultra centrifugal filter (30-kDa MWCO), and the resulting GLipo was collected. Encapsulation efficiency (%) was determined by quantifying the amount of gem entrapped within the liposomal core via UV–visible spectroscopy at 268 nm. The particle size (nm) and zeta potential (mV) of GLipo were assessed using a particle size analyzer (Zeta Potential Analyzer, ELSZ2000, Japan). The morphology of GLipo was investigated using transmission electron microscopy (JEM-1010, Jeol, Japan) at an accelerating voltage of 80 kV. Additionally, the stability of GLipo in both complete growth media and growth media was evaluated by monitoring changes in hydrodynamic particle size at multiple time points.

### NK membrane decoration with GLipo

To observe the attachment of liposomes onto NK cell surfaces via the DBCO–azide reaction, 5-carboxyfluorescein (Sigma-Aldrich, 86826)-loaded liposomes containing azide groups (DLipo) were prepared and incubated with either NK or DBCO-NK cells. Utilizing copper-free bio-orthogonal click chemistry, the azide functionalities on DLipo conjugated with the surface DBCO groups present on DBCO-NK cells. Following 2 sequential washing steps, NK cells incubated with DLipo without the click reaction (DLipo + NK; nondecorated control) and DLipo-NK cells (decorated via the click reaction) were visualized by fluorescence microscopy. These fluorescence images were subsequently analyzed using the Nikon NIS-AR software (ver. 4.40). For GLipo, the same procedure was applied and the quantity of gem conjugated on the NK cell surfaces was then determined. After GLipo attachment, NK cells were isolated by centrifugation, and the resulting cell pellets were subjected to lyophilization. The dry pellets were dissolved in a solvent mixture consisting of 15% ethyl acetate in methanol, followed by rigorous vortexing for 2 min. Samples were centrifuged at 13,000 rpm for 15 min, after which the supernatant was collected and the organic layer allowed to evaporate. The mobile phase, consisting of 10% acetonitrile containing a fixed concentration of theophylline (100 μM) as an internal standard, was added to each vial. Each sample was then filtered through a 0.2-μm syringe filter and analyzed using a high-performance liquid chromatography (HPLC) system for gem quantification. Quantification was performed using an HPLC instrument (Agilent 1100 series) fitted with a photodiode array detector and a Symmetry C18 column (Waters, 250 × 4.6 mm, 5 μm). The column temperature was set at 25 ± 5 °C, and the mobile phase comprised acetonitrile (10%) in water, pH adjusted to 7.0, maintained at a 1 ml min^−1^ flow rate. Theophylline was employed as the internal standard. Each sample injection volume was fixed at 20 μl; the retention time for gem was 3.4 min, and that for theophylline was 6.9 min. The calibration graph displayed linearity over the 0.5 to 100 μM concentration range, with a correlation coefficient of 0.999.

### Drug release analysis

The release profile of gem from the GLipo formulation was examined in buffer media of pH 5.0, 6.8, and 7.4 at 37 °C. A volume of 1 ml of GLipo was loaded into a dialysis bag (MWCO: 2 kDa), which was submerged in 10 ml of release buffer and incubated at 37 °C. At designated time points (0.5, 1, 3, 6, 12, 24, 48, 72, and 96 h), 100 μl of buffer was withdrawn and replaced with the same volume of fresh buffer to maintain constant volume. The percentage of released gem was quantified using a UV–visible spectrophotometer at 268 nm.

### In vitro anticancer assay

The anticancer efficacy of the GLipo-NK complex was evaluated using the WST-1 assay. Initially, the cytotoxic effects of GLipo on NK cells were determined. NK cells were seeded into 96-well plates (1.0 × 10^5^ per well) and exposed to a range of GLipo concentrations (0 to 100 μg/ml). After a 24-h incubation period, NK cell viability was assessed via the WST-1 assay according to the manufacturer’s instructions. To investigate therapeutic efficacy against cancer cells, a co-culture system was utilized with NK cells and cancer cells. First, MIA PaCa-2 cells were seeded at 1.0 × 10^5^ cells per well and incubated for 24 h. Subsequently, NK, Lipo-NK, and GLipo-NK cells (gem 87.5 ng/2.0 × 10^5^ NK cells) were introduced and co-cultured for an additional 24 h. After the incubation, supernatants were collected, and the anticancer efficacy was quantified by the WST-1 assay following the manufacturer’s protocol. The percentage of cancer viability was calculated using the following equation: Cancer viability (%) = [(experimental value − effector control)/(target cell spontaneous control)] × 100 (%), where the experimental value corresponds to co-cultures of NK or DBCO-NK cells with MIA-PaCa-2 cells [31].

### Statistical analysis

All quantitative data are presented as mean ± standard deviation and were analyzed by a *t* test and one-way analysis of variance using GraphPad Prism V 7.0 (GraphPad Software Inc., USA).

## Results and Discussion

### Synthesis and characterization of DBCO–lipid biomaterials for NK cell surface engineering

The synthesis of DBCO-functionalized lipid biomaterials is a crucial advancement for the development of sophisticated NK cell surface engineering methodologies. The incorporation of DBCO groups onto NK cell surfaces facilitates bio-orthogonal strain-promoted azide–alkyne cycloaddition reactions with other entities, enabling rapid, catalyst-free click conjugation under physiological conditions [32]. These biomaterials were applied to achieve targeted and durable functionalization of the cell surface membrane while maintaining cellular viability and phenotype. This approach offers a reliable and flexible platform for NK cell surface modification aimed at improving target specificity, therapeutic payload delivery, or immune cell tracking in cancer immunotherapy [33]. In our previous work, we optimized biomaterials mediated by diverse types of lipids (e.g., DSPE, DMPC, and cholesterol) for effective NK cell surface engineering [13]. DSPE-based biomaterials, recognized for their prominent hydrophobic interactions and prolonged membrane association, serve as highly suitable lipid candidates for the surface modification of NK cells. Their capacity for stable membrane integration ensures that conjugated functional groups effectively anchor to NK cell surfaces for extended durations [13]. Conversely, vitamin E lipid failed to achieve stable anchoring on NK cells, while nearly 80% of cholesterol-based material detached within 1 h of cell surface modification. Therefore, DSPE lipid demonstrated superior stability, making it the optimal choice of NK cell surface engineering owing to its strong hydrophobicity [34]. Accordingly, we selected DSPE as the lipid anchor for the synthesis of DBCO–lipid biomaterials.

**Fig. 3.**
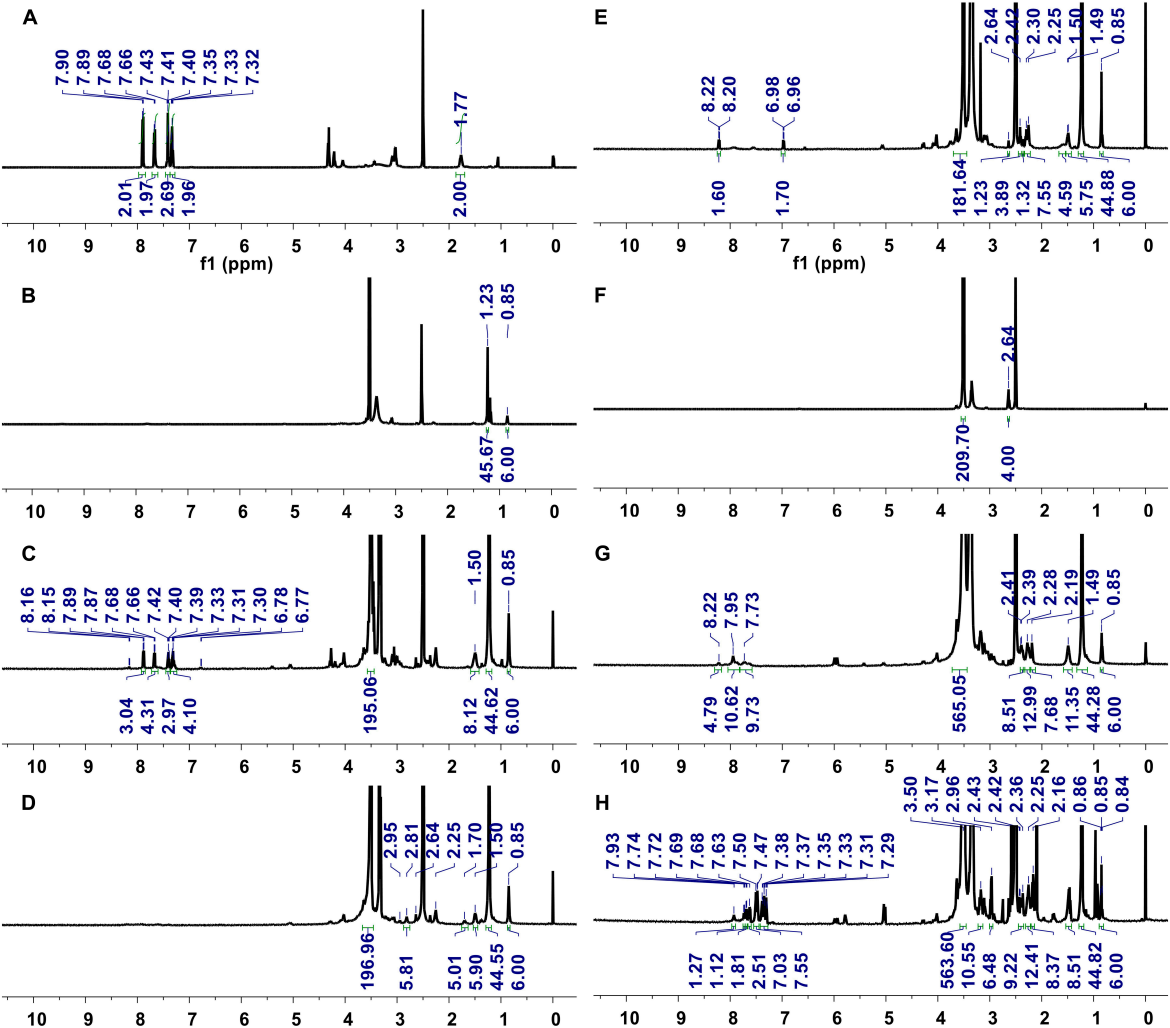
Proton nuclear magnetic resonance (NMR) spectra of the tadpole-style DSPE-PEG2k-Di-PEG2k-DBCO biomaterial. (A) ^1^H NMR spectrum (500 MHz, dimethyl sulfoxide-d_6_ [DMSO-d_6_]) of Di-Fmoc-Gly, (B) ^1^H NMR spectrum (500 MHz, DMSO-d_6_) of DSPE-PEG2k-amine, (C) ^1^H NMR spectrum (500 MHz, DMSO-d_6_) of DSPE-PEG2k-Gly-Di-Fmoc, (D) ^1^H NMR spectrum (500 MHz, DMSO-d_6_) of DSPE-PEG2k-Gly-Di-amine, (E) ^1^H NMR spectrum (500 MHz, DMSO-d_6_) of DSPE-PEG2k-Gly-Di-COOH, (F) ^1^H NMR spectrum (500 MHz, DMSO-d_6_) of bis-PEG-amine, (G) ^1^H NMR spectrum (500 MHz, DMSO-d_6_) of DSPE-PEG2k-Di-PEG, and (H) ^1^H NMR spectrum (500 MHz, DMSO-d_6_) of DSPE-PEG2k-D-PEG-DBCO biomaterial.

The various types of DBCO–lipid conjugates were synthesized as follows: (a) 2 linear constructs featuring different PEG chain lengths (DSPE-PEG2k-DBCO and DSPE-PEG5k-DBCO), (b) a branched structure (DSPE-PEG2k-HA-DBCO), and (c) a tadpole-like structure (DSPE-PEG2k-Di-PEG2k-DBCO). In the branched structure, HA was utilized as a polymeric backbone to facilitate the multivalent display of DBCO moieties, which in turn enhanced conjugation efficiency with azide-functionalized liposomes. The synthesis processes of linear-style DBCO conjugates are depicted in Fig. S1, and the molecular structures were verified by ^1^H NMR analysis. In Fig. S3, the NMR spectra of the synthesized linear DSPE-PEG-DBCO materials are presented. DSPE-PEG2k-DBCO demonstrates distinct ^1^H NMR (500 MHz, dimethyl sulfoxide [DMSO]) resonances including the terminal methyl group of DSPE (*δ* 0.85 ppm), PEG 2000 repeating units (*δ* 3.51 ppm), DBCO phenyl group protons (*δ* 7.29 to 7.93 ppm), and amide protons (*δ* 8.53 to 8.59 ppm) (Fig. S3A). The DSPE-PEG5k-DBCO sample displays characteristic ^1^H NMR (500 MHz, DMSO) resonances for the terminal methyl group of DSPE (*δ* 0.85 ppm), PEG 5000 repeating units (*δ* 3.51 ppm), and DBCO phenyl group protons (*δ* 7.29 to 7.76 ppm) (Fig. S3B). Likewise, the steps of the synthesis processes of the branch-style DSPE-PEG2k-HA-DBCO conjugate are depicted in Fig. S2, and the ^1^H NMR spectra of intermediate (DSPE-PEG-HA) and final material (DSPE-PEG2k-HA-DBCO) are represented in Fig. S4. DSPE-PEG2k-HA-DBCO exhibits ^1^H NMR (500 MHz, DMSO) peaks including DSPE lipid terminal methyl protons (*δ* 0.98 ppm), the *N*-acetyl COCH_3_ group of the HA backbone (*δ* 1.92 ppm), PEG protons from the lipid-PEG component (*δ* 3.51 ppm), and DBCO phenyl group protons (*δ* 7.15 to 7.91 ppm). The extent of lipid-PEG substitution was determined through the integration of NMR spectra using the signals at *δ* 1.92 ppm (*N*-acetyl COCH_3_ group) and *δ* 0.98 ppm (DSPE terminal CH_3_ groups). The integration results indicate that 10% of the HA backbone is substituted with lipid-PEG (Fig. S4). Furthermore, the steps of the synthesis processes of the tadpole-like synthesized DSPE-PEG2k-Di-PEG2k-DBCO are depicted in Fig. 2, the ^1^H NMR spectra of intermediates and the final DBCO conjugate represented in Fig. 3, and the proton signals of the synthesized intermediates are provided in Section S1 (Supplementary Materials). The ^1^H NMR (500 MHz, DMSO) signals of DSPE-PEG2k-Di-PEG2k-DBCO peaks include the DSPE terminal methyl group (*δ* 0.84 to 0.86 ppm), 2-lipid methyl chain protons (*δ* 2.16 to 2.96 ppm), PEG 2000 repeating units (*δ* 3.50 ppm), and DBCO phenyl group protons (*δ* 7.29 to 7.93 ppm). Proton integration analysis based on the DSPE terminal methyl protons at *δ* 0.85 ppm (6H), compared to PEG protons at *δ* 3.50 ppm (563H), along with the presence of DBCO phenyl signals confirms successful synthesis of DSPE-PEG2k-Di-PEG2k-DBCO.

### Optimization of NK cell surface engineering with different biomaterials

**Fig. 4.**
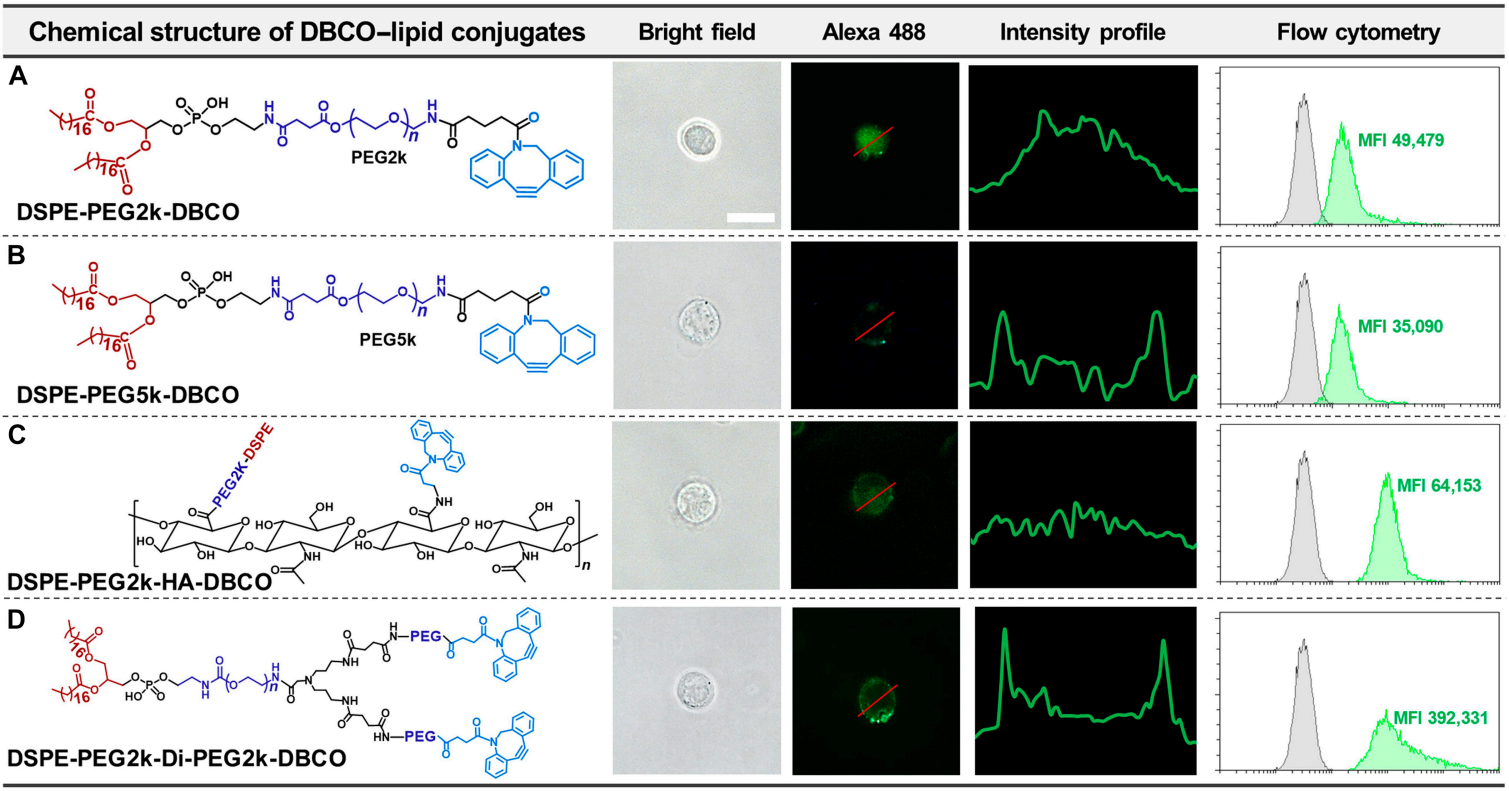
Optimization of DBCO–lipid based on fluorescence imaging following NK cell surface engineering. The lipid anchor was unified to DSPE, and the following variants were tested: (A) DSPE-PEG2k-DBCO, (B) DSPE-PEG5k-DBCO, (C) DSPE-PEG2k-HA-DBCO, and (D) DSPE-PEG2k-Di-PEGc2k-DBCO. NK cells were exposed to various DBCO–lipid conjugates and incubated for 30 min at room temperature (RT). To detect DBCO presentation on cell membranes, BP Fluor 488 Picolyl Azide was applied, and fluorescence images were obtained using fluorescence microscopy. The mean fluorescence intensity (MFI) of NK cells coated with different DBCO–lipid conjugates was further evaluated by flow cytometry.

To assess successful membrane anchoring, the surface presentation of DBCO moieties on NK cell membranes was examined. For optimization, DBCO–lipid conjugates with distinct structural forms were designed, including DSPE-PEG2k-DBCO (linear PEG2k), DSPE-PEG5k-DBCO (linear PEG5k), DSPE-PEG2k-HA-DBCO (branched PEG2k), and DSPE-PEG2k-Di-PEG2k-DBCO (tadpole structure) (Fig. 4). Fluorescence imaging after labeling with BP Fluor 488 Picolyl Azide (Fluor 488-N_3_) revealed that DSPE-PEG2k-DBCO (linear PEG2k) and DSPE-PEG2k-HA-DBCO (branched PEG2k) showed limited membrane localization with increased cytoplasmic internalization (Fig. 4A and C). In contrast, DSPE-PEG5k-DBCO (linear PEG5k) and DSPE-PEG2k-Di-PEG2k-DBCO (tadpole structure) exhibited a uniform membrane coating after 30 min of incubation (Fig. 4B and D). On this basis, the tadpole structure DSPE-PEG2k-Di-PEG2k-DBCO was selected for further study owing to its superior coating efficiency on NK cell membranes. The improved surface presentation of DSPE-PEG2k-Di-PEG2k-DBCO is likely due to its optimized amphiphilic balance, facilitating enhanced hydrophobic interactions with NK cell membranes [14]. Moreover, enhanced DBCO exposure was confirmed by flow cytometry, where NK cells coated with DSPE-PEG2k-Di-PEG2k-DBCO exhibited the highest mean fluorescence intensity (MFI), while those coated with DSPE-PEG2k-DBCO, DSPE-PEG5k-DBCO, and DSPE-PEG2k-HA-DBCO displayed comparatively lower MFIs (Fig. 4B and D). In contrast, both the PEG2k linear and branched structures exhibited substantially lower membrane anchoring efficiency. For PEG2k linear variants, the short PEG linker insufficiently exposed the DBCO moieties, which likely contributed to an increased tendency for internalization into the cytoplasm. As shown in Fig. S5, fluorescence microscopy images of NK cells incubated with Fluor 488-N_3_ alone showed intracellular fluorescence patterns consistent with those observed in the groups displaying material internalization shown in Fig. 4A and C.

In the case of the branched structure, steric hindrance arising from the substantial HA backbone hindered effective membrane insertion and limited the accessibility of azide to DBCO groups. Regarding DSPE-PEG2k-HA-DBCO, elevated local concentrations of PEG-lipid can facilitate self-folding or aggregation, whereas insufficient concentrations lead to poor membrane anchoring because of weak hydrophobic interactions with the NK membrane. Collectively, for effective NK surface modification with DBCO–lipid anchoring materials, it is critical to minimize steric hindrance and ensure that the DBCO group is adequately exposed to enable high surface attachment of drug-loaded liposomes in subsequent steps. In this context, bulky backbone-based constructs are suboptimal, as they impede efficient liposome conjugation on NK cell surfaces. Thus, the tadpole-structured DBCO–lipid conjugate was identified as the optimal candidate based on the following criteria: (a) streamlined molecular structure, (b) minimized structural hindrance, and (c) enhanced capability for DBCO exposure.

### Distinctive features of DBCO-engineered NK cells

**Fig. 5.**
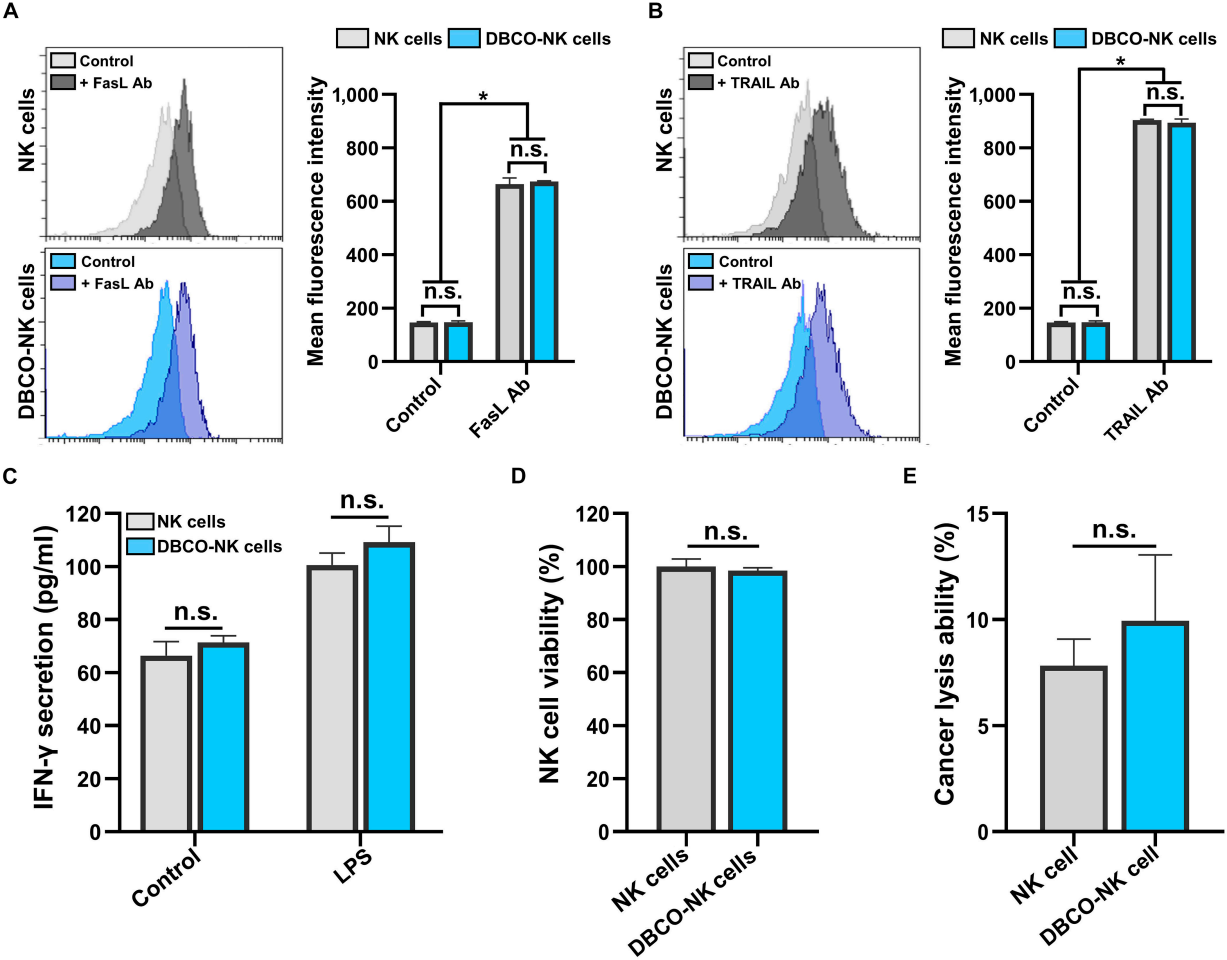
Evaluation of the intrinsic properties of DBCO-NK cells. (A and B) Surface expression of native death ligands Fas ligand (FasL) and tumor-necrosis-factor-related apoptosis-inducing ligand (TRAIL) on DBCO-NK cells, determined by flow cytometry; (C) interferon-γ (IFN-γ) secretion in response to lipopolysaccharide stimulation, quantified by enzyme-linked immunosorbent assay (ELISA) from NK cells and DBCO-NK cells; (D) viability of NK cells coated with DSPE-PEG-Di-PEG-DBCO after 24-h incubation, measured by the WST-1 assay; and (E) cytotoxicity of NK cells coated with DSPE-PEG2k-Di-PEG2k-DBCO against cancer cells, assessed after 24-h co-culture at a 2:1 effector-to-target (E:T) ratio, measured by the WST-1 assay. * indicates statistical significance compared with the NK cell group (*P* < 0.05); n.s. indicates no significant difference between groups. Ab, antibody; LPS, lipopolysaccharide.

As the NK cell membrane plays a crucial role in forming immunological synapses and recognizing target tumor cells through the interaction of surface ligand/receptor compartments, it was essential to confirm that the native intrinsic functions of NK cells were preserved after coating [35]. Accordingly, several membrane-associated properties were assessed, including surface ligand availability, cytokine secretion following antigenic stimulation, cell viability, and intrinsic anticancer activity (Fig. 5). DBCO–lipid conjugates enabled efficient cell surface coating by exploiting hydrophobic interactions between lipid moieties and the NK cell membrane during a straightforward 30-min incubation at RT (Fig. 5A and B). For effective NK-cell-mediated recognition and killing of cancer cells, FasL and TRAIL serve as key cytotoxic mediators present on NK membranes [36]. To verify that these native ligands remained available after coating, either APC-conjugated FasL antibody (Fig. 5A) or APC-conjugated TRAIL antibody (Fig. 5B) was used, and the surface presence of both ligands was analyzed by flow cytometry. The comparable MFIs observed between unmodified and DBCO-coated NK cells demonstrated that the DBCO–lipid conjugate coating process did not mask or interfere with ligand presentation and interaction with other molecules. As an additional evaluation of the immunological signaling pathway, IFN-γ secretion after LPS stimulation [13,14] was also determined (Fig. 5C). Both NK and DBCO-NK cells displayed similar IFN-γ secretion levels. LPS, a representative pathogen-associated molecular pattern [16,37], binds directly to Toll-like receptor 4 (TLR4) on NK cell membranes, initiating intracellular signaling events that result in IFN-γ production. After receptor binding, the LPS–TLR4 complex is internalized, triggering downstream pathways and ultimately facilitating IFN-γ secretion through the cell membrane. Our results showed that engineering NK cell surfaces with DBCO–lipid conjugates did not alter (a) signal binding to membrane receptors, (b) intracellular signal relay, (c) cytokine expression, or (d) the exocytosis of the produced molecules across the membrane.

Subsequently, the effects of DBCO–lipid conjugates on both NK cell viability (Fig. 5D) and cytotoxic function (Fig. 5E) were evaluated. NK cell viability remained consistent over 24 h, irrespective of the presence of DBCO–lipid conjugates (Fig. 5D). Additionally, both NK and DBCO-NK cells exhibited similar intrinsic cytotoxic activity against pancreatic cancer cells (i.e., MIA PaCa-2) over the same period, without a statistically significant difference (Fig. 5E). As further illustrated in Fig. 5A and B, this cytotoxicity results from NK–cancer membrane interactions, particularly through the engagement of FasL and TRAIL with corresponding cancer surface antigens. Overall, the retained cytotoxic efficacy of coated NK cells toward target MIA PaCa-2 demonstrated that NK surface modification via DBCO–lipid conjugates preserves stepwise immunological functions, ranging from surface ligand display to cancer cell killing.

### Fabrication and characterization of GLipo

For efficient conjugation with surface-immobilized DBCO on NK cell membranes, azide-functionalized liposomes were intentionally engineered via strain-promoted azide–alkyne cycloaddition. These liposomes were produced using thin-film hydration by incorporating DSPE-PEG2k-azide into the DMPC lipid bilayer, with gem encapsulated in the liposomal core (GLipo). The surface-displayed azide groups enable rapid, catalyst-free covalent binding with DBCO moieties on the NK cell surface, leading to site-specific decoration with the engineered liposomes. This strategy enables the CDC formation of gem-loaded liposome–NK cell hybrids (GLipo-NK) for targeted delivery of gem while preserving NK cell viability and functional activity, thereby facilitating advanced codelivery of chemo-drug employing immune cell carriers.

**Fig. 6.**
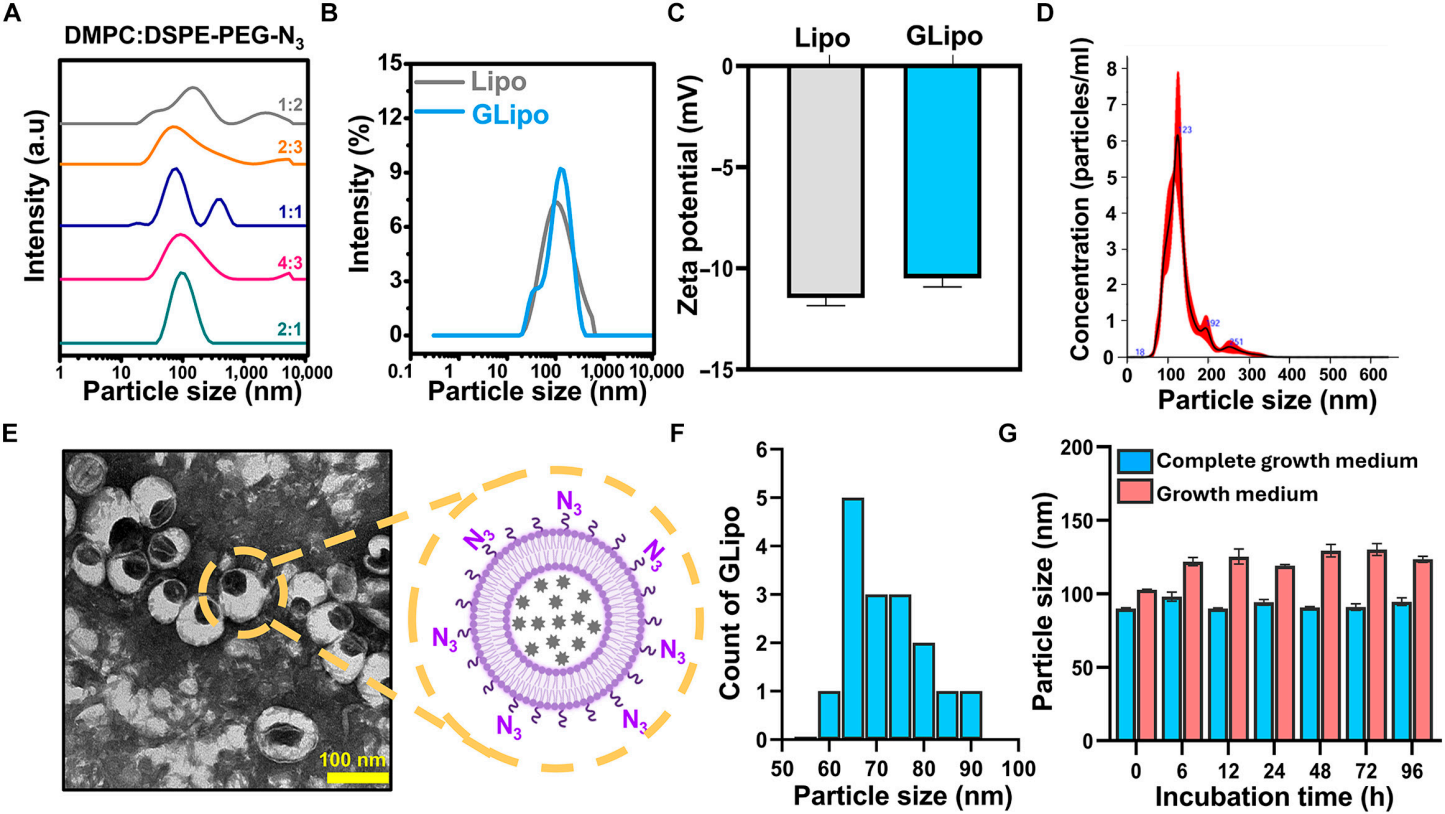
GLipo fabrication and characterization. (A) Particle size distributions of various ratios of lipid and lipid-PEG, (B) particle size distributions of optimized liposomes (Lipo) and GLipo, (C) zeta potentials of optimized Lipo and GLipo, (D) nanoparticle tracking analysis (NTA) of GLipo, (E) transmission electron microscopy (TEM) image of GLipo, (F) particle size distribution of GLipo determined from the TEM image using the ImageJ software, and (G) evaluation of GLipo stability via the measurement of particle size (using dynamic light scattering [DLS]) in the complete growth medium and growth medium at different time intervals.

To achieve highly stable liposomes with a uniform particle size distribution, various combinations of DMPC and DSPE-PEG2k-azide were evaluated by adjusting their molar ratios to identify an optimal formulation that preserves the integrity of the liposomal membrane while allowing maximal gem loading. The 2:1 ratio of DMPC:DSPE-PEG-azide produced the most favorable results, yielding a uniform particle size of 97.99 ± 1.4 nm and a low polydispersity index of 0.186 ± 0.02 (Fig. 6A and Table S1). These optimized liposomes exhibited a stable zeta potential of −11.47 ± 0.35 mV, confirming good colloidal stability and reduced aggregation. Following the loading of gem into the liposomes (GLipo), the particle size increased slightly to 132.6 ± 2.2 nm (Fig. 6B), with a polydispersity index of 0.203 ± 0.01 and a zeta potential of −10.49 ± 0.4 mV (Fig. 6C) [38]. Nanoparticle tracking analysis of the optimized liposomes indicated a single peak with a mean size of 132.5 ± 5.3 nm with a particle concentration of 4.73 × 10^8^ ± 1.61 × 10^7^ particles/ml (Fig. 6D). Transmission electron microscopy revealed the spherical morphology of the GLipo formulation, with an approximate particle size of 71.5 ± 7.9 nm (Fig. 6E and F) [39]. The encapsulation efficiency of gem within the liposomes was determined to be 81.5% ± 1.6%, indicating efficient drug loading. In addition, the stability of GLipo was assessed in both complete growth medium and growth medium, and the particle size remained unchanged after 96 h of incubation (Fig. 6G). These findings indicate that the optimized GLipo formulation demonstrates excellent stability and is well suited for immobilization onto DBCO-modified NK cells, facilitating the creation of GLipo-NK therapeutic constructs.

### Covalent attachment of GLipo onto surface-modified NK cells

**Fig. 7.**
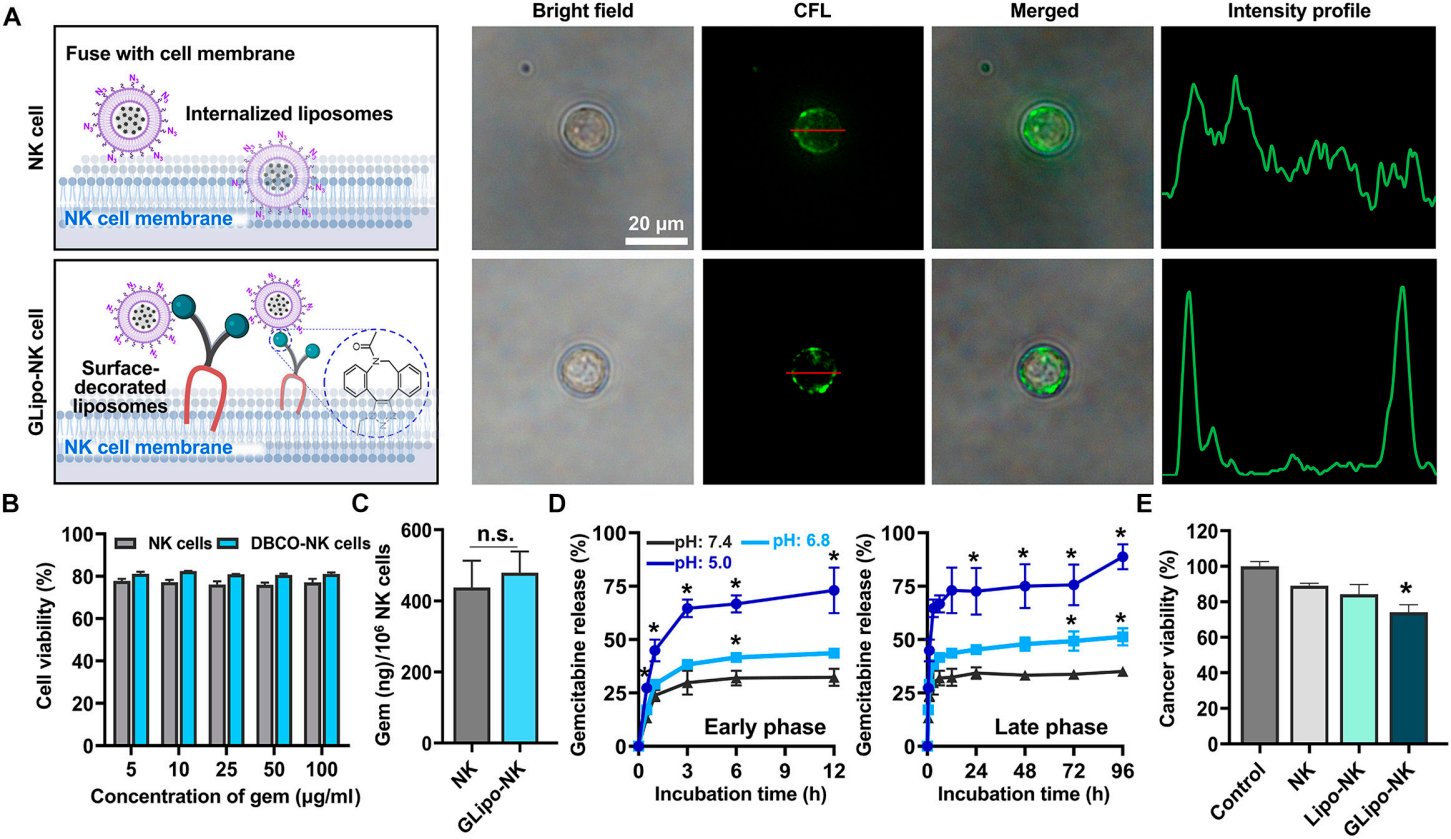
(A) Schematic illustration of liposome attachment to both NK cells and DBCO-NK cells, as well as fluorescence images showing dye-loaded liposomes (DLipo) anchoring to noncoated NK cells and DBCO-NK cells; (B) viability of NK cells and GLipo-NK cells; (C) quantification of gem immobilized on NK and DBCO-NK cells; and (D) in vitro drug release behavior of GLipo in buffer solutions at pH 5.0, pH 6.8, and pH 7.4. * indicates a statistically significant difference compared with the release profile at pH 7.5 for each time point (*P* < 0.05). (E) Cancer viability resulting from GLipo-NK cell treatment. NK cells, Lipo-NK, or GLipo-NK cells were co-cultured with target cells using a 2:1 E:T ratio at 37 °C for 4 h. * indicates a statistically significant difference compared to the NK cell group (*P* < 0.05). CFL, carboxyfluorescein.

Surface-modified NK cells with DBCO–lipid conjugates were subsequently used to incorporate GLipo through covalent conjugation between the surface-presented azide groups and GLipo, employing bio-orthogonal click chemistry (Fig. 7A). Specifically, the azide–alkyne cycloaddition, a catalyst-free reaction, enabled site-specific and stable covalent attachment of GLipo onto the DBCO-NK cell membrane while preventing cellular internalization. The covalent immobilization of GLipo ensured localized and sustained gem release at tumor sites and maintained the natural tumor-targeting features of NK cells. To optimize the conjugation process, incubation time was evaluated to determine the sufficient period for bio-orthogonal click chemistry. An incubation period of 30 min was identified as sufficient for effective conjugation between an azide-based labeling dye and DBCO-engineered NK cells (Fig. S6) [40,41]. To confirm the membrane incorporation of GLipo by DBCO–azide conjugation, dye-loaded liposomes (DLipo) were generated using the same protocol as GLipo fabrication. The fluorescence images in Fig. 7A display the colocalization of DLipo with DBCO-NK cells, providing clear evidence of successful surface conjugation. In contrast, noncoated NK cells exhibited partial DLipo internalization or membrane fusion. These results demonstrate the effectiveness of our DBCO–lipid anchor-based surface engineering for producing CDCs that achieve sufficient membrane incorporation of chemo-drug-loaded liposomes without undesired cytoplasmic internalization. To further confirm that liposome treatment does not adversely affect NK cell viability, WST-1 assays were performed with NK cells and DBCO-NK cells treated with varying numbers of Lipo, indicating no significant differences in cell viability across conditions (Fig. S7). The impact of GLipo on NK cell viability was next assessed following treatment with varying concentrations of encapsulated gem (Fig. 7B). GLipo treatment did not markedly impact the viability of either NK or DBCO-NK cells at different gem concentrations within the liposomes. By comparison, cell viability was significantly reduced in both NK and DBCO-NK cells upon administration of bolus gem. These data indicate that encapsulation of gem in liposomes enables safer drug delivery through NK cell carriers, protecting their viability while supporting targeted anticancer efficacy. Following the incubation of NK and DBCO-NK cells with GLipo, the quantification of gem localized on the cell surface revealed values of 479.78 ± 59.38 and 437.63 ± 75.75 ng/million NK cells for noncoated NK cells and DBCO-NK cells, respectively (Fig. 7C).

### Exploring controlled drug release profiles and improved anticancer efficacy

The successful development of CDCs using GLipo confirms that the conjugation strategy preserves the functional viability of carrier NK cells. After homing to the TME, CDCs enable site-specific release of gem in response to environmental signals such as acidic pH, enzymatic activity, or membrane fusion events [42,43]. This site-selective release reduces systemic toxicity to normal tissues while maintaining an elevated local gem concentration at the tumor site, thus strengthening cytotoxic efficacy. Additionally, NK cells facilitate tumor elimination through their inherent cell-mediated cytotoxic activity [44]. Collectively, the combination of spatially controlled drug release and active immunosurveillance markedly improves anticancer outcomes. To evaluate the pH-dependent release characteristics of GLipo, in vitro gem release assays were performed at 37 °C across a range of pH levels: physiological pH 7.4, TME-like pH 6.8, and endosomal pH 5.0. At pH 7.4, a diminished release of ~35% was detected, reflecting the structural integrity of GLipo under physiological conditions and thereby minimizing the likelihood of premature drug loss. At slightly acidic pH 6.8, simulating the TME, the release increased to ~51%, with a pronounced release of ~90% at endosomal pH 5.0 (Fig. 7D). These findings clearly demonstrate GLipo’s pH-responsive release profile, characterized by limited drug release during systemic circulation and robust drug delivery in acidic tumor settings. The biphasic release pattern of gem observed in Fig. 7D can be divided into two distinct phases. The initial early phase (0, 3, 6, 9, and 12 h) reflects a fast release profile under a slightly acidic TME, thereby allowing immediate therapeutic activity against cancer cells. This burst release ensures sufficient drug availability to trigger an early anticancer effect. In contrast, the late phase (24, 48, 72, and 96 h) corresponds to sustained release at endosomal acidic pH 5.0, which prolongs intracellular drug exposure and enhances cytotoxicity by reducing cancer cell viability. Such sustained kinetics are biologically advantageous, as they help maintain effective drug concentrations within the TME while supporting prolonged therapeutic efficacy [45]. In summary, this pH-triggered release property substantiates the potential of CDCs to augment NK-cell-driven anticancer efficacy via site-specific and regulated drug delivery.

The NK-cell-mediated anticancer response initiates with targeted interactions at the cancer cell membrane and subsequent immunological synapse formation, which stimulates NK cells to secrete cytotoxic granules, including perforin and granzyme, ultimately resulting in the destruction of cancer cells. Analogously, the anticancer efficacy of engineered NK cells is enhanced by improved cell–cell contacts, thereby increasing their capability to eradicate solid tumors. The stepwise anticancer mechanism of engineered NK cells consists of (a) recognition of target cells, (b) activation of immune cells, and (c) induction of cancer cell death [13]. Building on this mechanism, we propose a foundational approach to developing a chemo-drug-immobilized, cell-based therapeutic system, termed CDC. This strategically engineered, cell-based drug delivery platform demonstrated successful application for targeted cancer cell elimination. The in vitro anticancer effectiveness of CDCs was examined by conducting a cancer cell viability assay. As presented in Fig. 7E, at a 2:1 effector-to-target ratio, naïve NK cells maintained approximately 89% cancer cell viability, while Lipo-NK cells (not loaded with gem) resulted in about 84% viability, indicating only a slight effect due to liposome modification. In contrast, GLipo-NK application markedly reduced cancer cell viability to approximately 74%, demonstrating the enhanced therapeutic impact of the GLipo-NK CDC construct. These anticancer findings are consistent with our proposed mechanism: (a) establishment of cell–cell interaction between NK and cancer cells, (b) release of gem from the GLipo-NK construct, and (c) increased cancer cell death attributable to the delivered gem, specifically targeting pancreatic cancer cells.

## Conclusion

In summary, this study introduces an advanced strategy for improving NK-cell-based immunotherapy via precise ex vivo surface engineering with DBCO–lipid biomaterials and subsequent immobilization of GLipo. The synthesized lipid biomaterials include (a) 2 linear structures with distinct PEG chain lengths (DSPE-PEG2k-DBCO and DSPE-PEG5k-DBCO), (b) a branched structure (DSPE-PEG2k-HA-DBCO), and (c) a tadpole structure (DSPE-PEG2k-Di-PEG2k-DBCO). The research demonstrates (a) effective NK cell surface coating with multiple DBCO–lipid conjugate variants through membrane integration and (b) the generation of GLipo-NK cells by combining these with azide-functionalized GLipo. The resulting GLipo-NK cells exhibited potentiated anticancer effects via a combined mechanism of immune-mediated cytotoxic response and chemotherapeutic delivery. This methodology provides a nongenetic, modular, and scalable alternative to conventional CAR-based cell engineering approaches. Therefore, GLipo-NK cells developed using lipid-mediated surface engineering hold significant promise as cell-based therapies for targeting solid tumors, including pancreatic cancer.

## Data Availability

The datasets generated and/or analyzed during the current study are available from the corresponding author upon reasonable request.

## Supplementary Materials

Supplementary 1 Figs. S1 to S7 Table S1 Section S1
