# Supplementary material for: Development of Natural Killer Cell–Drug Conjugates via Membrane-Installed Liposomes for Pancreatic Cancer Treatment
Source: Biomater Res. 2025 Dec 9;29:0285. doi: 10.34133/bmr.0285 (PMC12686341; doi:10.34133/bmr.0285)
Supplement: Supplementary 1 — Figs. S1 to S7 Table S1 Section S1 [file bmr.0285.f1.docx]

**Supplementary information**

**Development of NK Cell-Drug Conjugates via Membrane-installed Liposomes for Pancreatic Cancer Treatment**

Ashok Kumar Jangid^1$^, Chae Eun Lee^1$^, Minseon Ryu^1^, Sungjun Kim^1^, and Kyobum Kim^1*^

^1^Department of Chemical & Biochemical Engineering, Dongguk University, 30, Pildong-ro 1-gil, Jung-gu, Seoul, Republic of Korea

^$^Authors contributed equally.

*Corresponding author:

Prof. Kyobum Kim ([kyobum.kim@dongguk.edu](mailto:kyobum.kim@dongguk.edu))

**
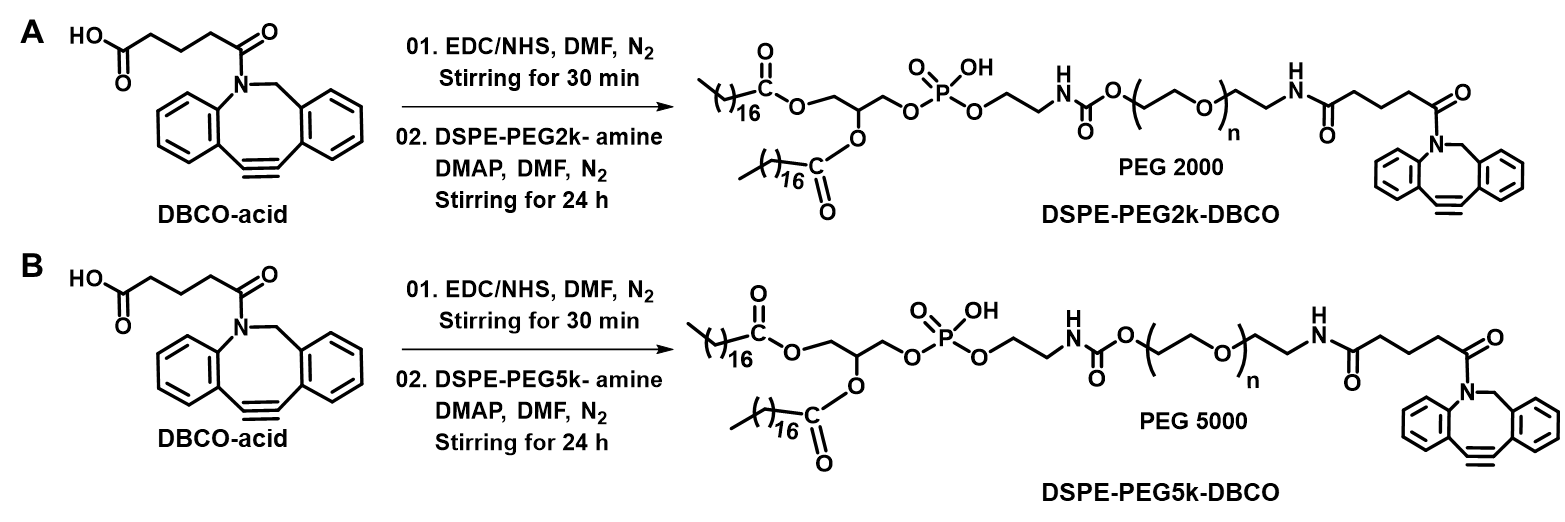
**

Fig. S1. Synthetic route for linear style (A) DSPE-PEG2k-DBCO and (B) DSPE-PEG5k-DBCO biomaterials.

**
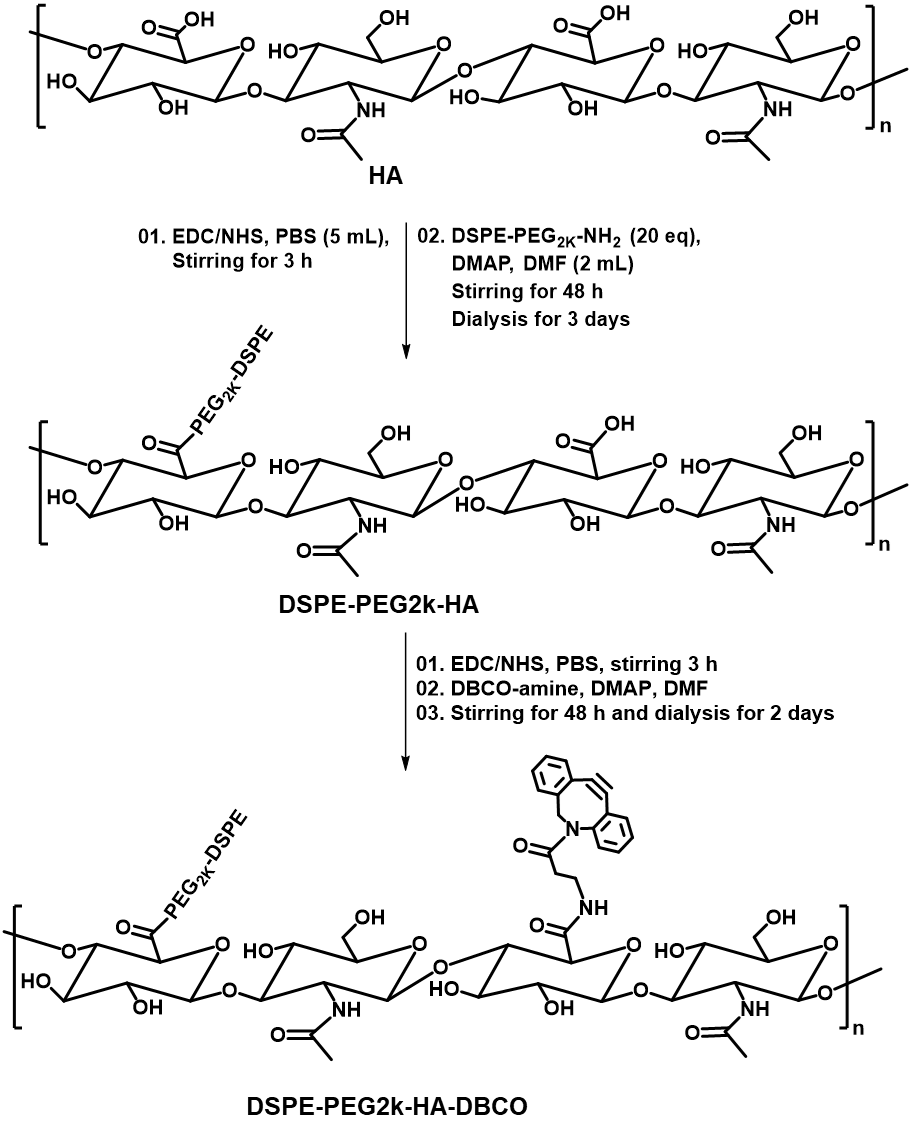
**

Fig. S2. Synthetic route for multibranch style DSPE-PEG2k-HA-DBCO biomaterial.


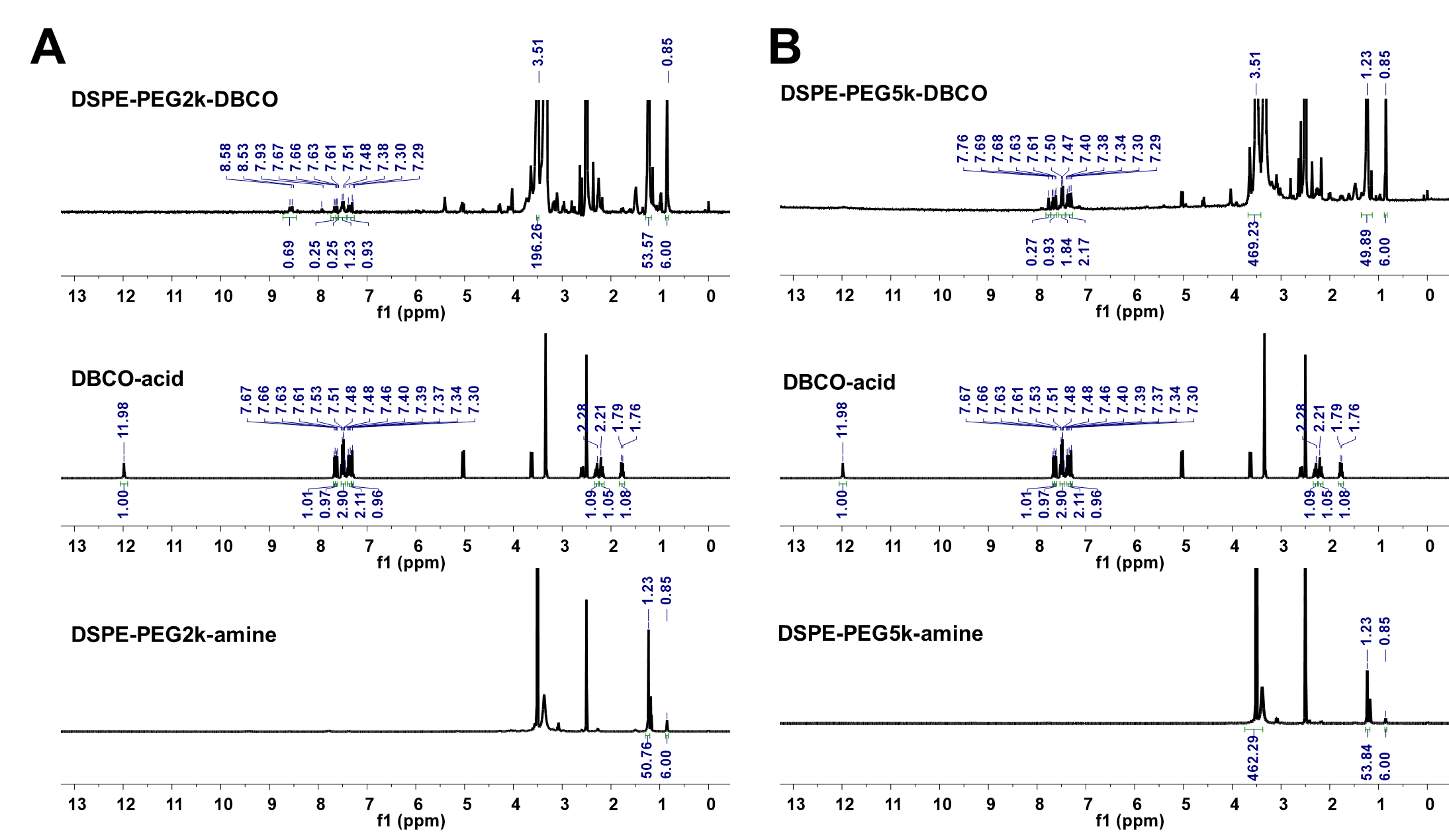


Fig. S3. NMR spectra of linear style DBCO-lipid biomaterials. (A) ^1^H NMR spectra (500 MHz, DMSO-d^6^) DSPE-PEG2k-amine, and DSPE-PEG2k-DBCO biomaterial, and (B) ^1^H NMR spectra (500 MHz, DMSO-d^6^) DSPE-PEG5k-amine, and DSPE-PEG5k-DBCO biomaterial.

**
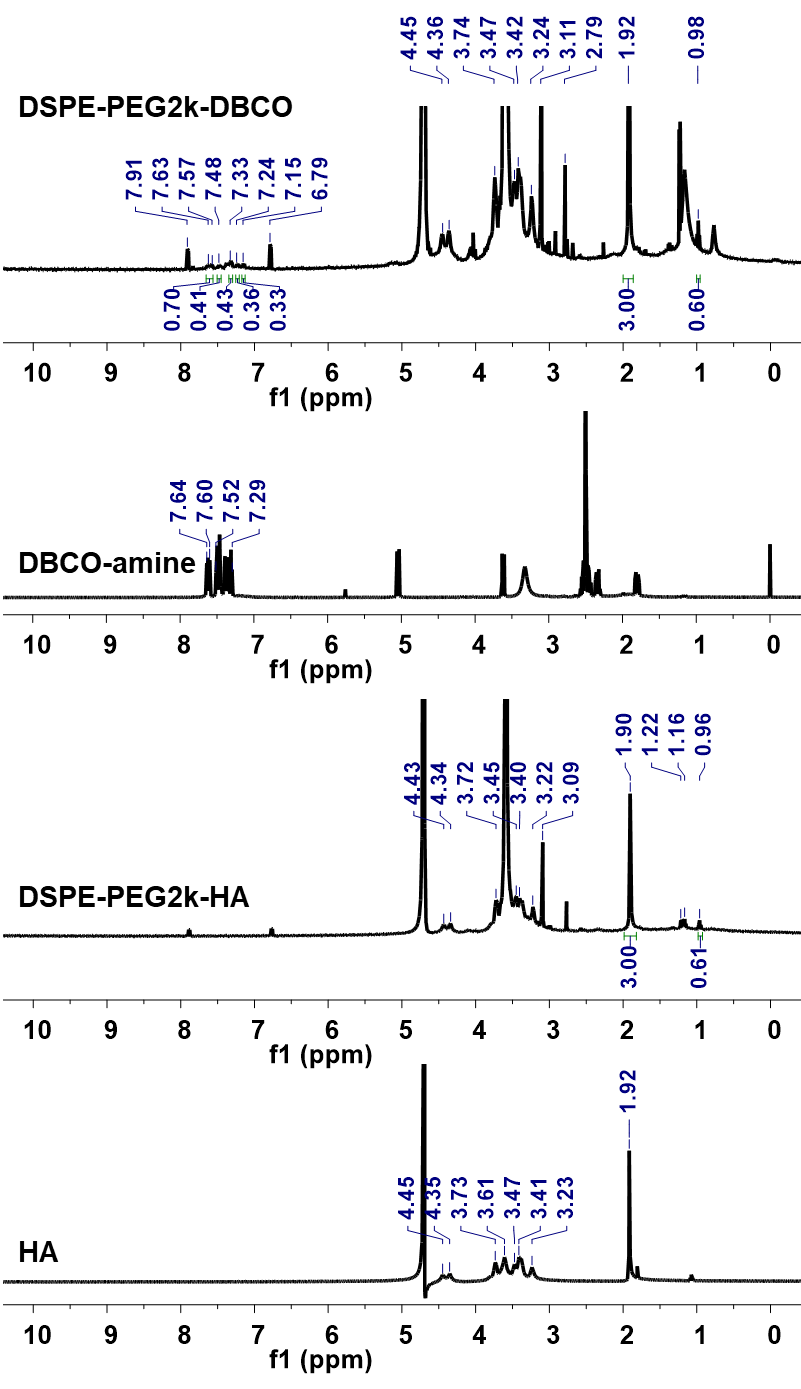
**

Fig. S4. NMR spectra of multibranch style DSPE-PEG2k-HA-DBCO biomaterial. ^1^H NMR spectra (500 MHz) of hyaluronic acid (HA) in D_2_O, DSPE-PEG conjugated HA intermediate (DSPE-PEG-HA) in D_2_O, DBCO-amine in DMSO-d6, and DBCO conjugated with DSPE-PEG-HA biomaterial (DSPE-PEG2k-HA-DBCO) in D_2_O.

**
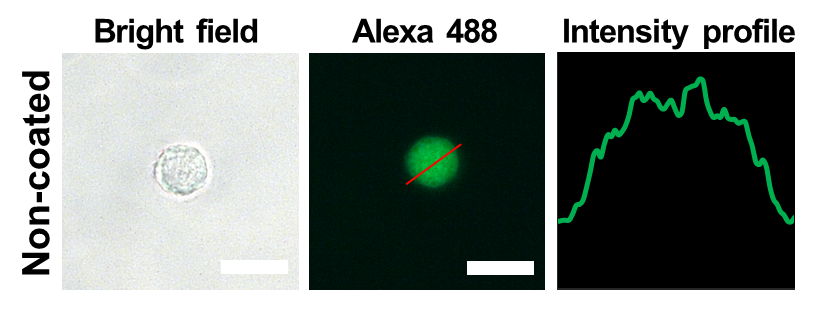
**

Fig. S5. Fluorescence microscopy image of NK cell treated with BP Fluor trea488 Picolyl Azide. (Scale bar: 10 μm.)


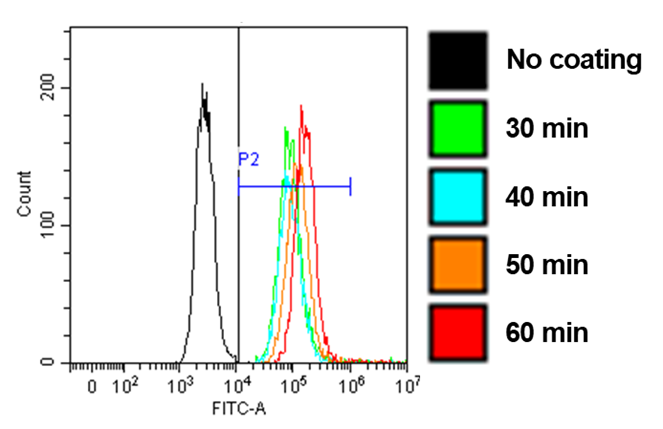


Fig. S6. MFI of NK cells coated with DSPE-PEG2k-Di-PEG2k-DBCO after BP Fluor488 Picolyl Azide treatment at different incubation times (30, 40, 50 and 60 min).


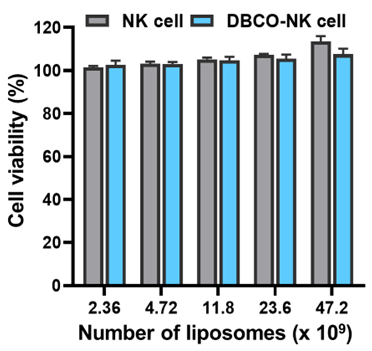


Fig. S7. WST-1 assay results for cell viability of NK cells and DBCO–NK cells following blank liposome treatment (without gemcitabine). A total of 4.72 x 10^9^ of liposomes corresponds to the number of liposomes treated at a GLipo concentration of 10 μg/mL in Fig. 7B.

**Table S1**. Particles sizes (nm) and polydispersity index (PDI) of different DMPC:DSPE-PEG2k-azide compositions.

| **DMPC: DSPE-PEG2k-azide** | **Size (nm) ± SD** | **PDI ± SD** |
| --- | --- | --- |
| 2:1 | 97.99 ± 1.41 | 0.186 ± 0.020 |
| 4:3 | 99.72 ± 1.49 | 0.337 ± 0.011 |
| 1:1 | 95.95 ± 4.15 | 0.397 ± 0.042 |
| 2:3 | 94.09 ± 4.86 | 0.427 ± 0.072 |
| 1:2 | 115.9 ± 1.46 | 0.597 ± 0.017 |

**Section S1**. DSPE-PEG2k-Di-PEG2k-DBCO intermediates ^1^H-NMR signals in ppm.

1. Gly-Di-Fmoc: 1H NMR (500 MHz, DMSO) δ 7.90, 7.89, 7.68, 7.66, 7.43, 7.41, 7.40, 7.35, 7.33, 7.32, 1.77.
2. DSPE-PEG-Gly-Di-Fmoc: 1H NMR (500 MHz, DMSO) δ 8.16, 8.15, 7.89, 7.87, 7.68, 7.66, 7.42, 7.40, 7.39, 7.33, 7.31, 7.30, 6.78, 6.77, 1.50, 0.85.
3. DSPE-PEG-Gly-Di-amine: ^1^H NMR (500 MHz, DMSO) δ 2.95, 2.81, 2.64, 2.25, 1.70, 1.50, 0.85.
4. DSPE-PEG-Gly-Di-COOH: ^1^H NMR (500 MHz, DMSO) δ 8.22, 8.20, 6.98, 6.96, 2.64, 2.42, 2.30, 2.25, 1.50, 1.49, 0.85.
5. DSPE-PEG-Gly-Di-PEG: ^1^H NMR (500 MHz, DMSO) δ 8.22, 7.95, 7.73, 3.50, 2.41, 2.39, 2.28, 2.19, 1.49, 0.85.
6. DSPE-PEG-Gly-Di-PEG-DBCO: ^1^H NMR (500 MHz, DMSO) δ 7.93, 7.74, 7.72, 7.69, 7.68, 7.63, 7.50, 7.47, 7.38, 7.37, 7.35, 7.33, 7.31, 7.29, 3.50, 3.17, 2.96, 2.43, 2.42, 2.36, 2.25, 2.16, 0.86, 0.85, 0.84.
